# Identifying 2SLGBTQ+ individuals experiencing homelessness using Point-in-Time counts: Evidence from the 2021 Toronto Street Needs Assessment survey

**DOI:** 10.1371/journal.pone.0298252

**Published:** 2024-04-10

**Authors:** Alex Abramovich, Max Marshall, Christopher Webb, Nicole Elkington, Rowen K. Stark, Nelson Pang, Linda Wood

**Affiliations:** 1 Institute for Mental Health Policy Research, Centre for Addiction and Mental Health, Toronto, Ontario, Canada; 2 Dalla Lana School of Public Health, University of Toronto, Toronto, Ontario, Canada; 3 Department of Psychiatry, University of Toronto, Toronto, Ontario, Canada; 4 Shelter, Support, and Housing Administration, City of Toronto, Toronto, Ontario, Canada; 5 Factor Inwentash Faculty of Social Work, University of Toronto, Toronto, Ontario, Canada; Universidad del Desarrollo, CHILE

## Abstract

**Introduction:**

The objective of this study was to utilize the data generated by the City of Toronto, Street Needs Assessment conducted in 2021 to explore the prevalence, causes, experiences, and characteristics of 2-spirit, lesbian, gay, bisexual, transgender, queer, and questioning (2SLGBTQ+) individuals experiencing homelessness in Toronto, Ontario, Canada.

**Methods:**

Data was collected by the City of Toronto during its Street Needs Assessment in April 2021. The Street Needs Assessment is a needs assessment survey and Point-in-Time count of people experiencing homelessness across the city of Toronto. Homelessness included any individual who was sleeping outdoors or staying in City-administered emergency/transitional shelters and shelter motels/hotels on the night of data collection. The Street Needs Assessment survey was administered to clients by trained shelter and outreach staff using a computer or mobile device. To ensure that survey questions were 2SLGBTQ+ inclusive, questions on sexual orientation, gender identity, and 2SLGBTQ+ identity were included in the survey.

**Results:**

Two hundred and eighty-eight 2SLGBTQ+ individuals completed the survey. Compared to non-2SLGBTQ+ individuals experiencing homelessness, 2SLGBTQ+ respondents were younger at the time of survey completion and when they first experienced homelessness, were more likely to have been in foster care or a group home, reported higher rates of conflict with and/or abuse by a parent/guardian as their main pathway into homelessness, and were more likely to experience chronic homelessness.

**Conclusion:**

Our study results demonstrate that Street Needs Assessments and Point-in-Time counts can be used to examine homelessness in marginalized populations, including 2SLGBTQ+ individuals and that sexual orientation and gender identity questions need to be included on future government surveys. The consistency of findings from this study and previous research suggests that 2SLGBTQ+ individuals experience a significant need for population-based housing and social support services aimed at meeting the needs of 2SLGBTQ+ populations.

## Background

Homelessness is a complex societal issue affecting diverse populations, each with their unique experiences and needs. Two-spirit, lesbian, gay, bisexual, transgender, queer, and questioning (2SLGBTQ+) individuals are disproportionately represented among people experiencing homelessness; however, accurate data on the prevalence of 2SLGBTQ+ individuals, particularly adults, experiencing homelessness in North America are lacking due to inadequate research and government prioritization of large-scale data collection among this population. The acronym 2SLGBTQ+ is used to refer collectively to sexual and gender minority individuals and is meant to be inclusive of the wide diversity of sexual and gender identities. Two-spirit refers to Indigenous people who identify as having both a masculine and a feminine spirit. This term is not exclusive to gender identity, and can also refer to sexual orientation, and spiritual identity. The term is a translation of the Anishinaabemowin term niizh manidoowag, two spirits [1].

Most research on homelessness among 2SLGBTQ+ populations that does exist in Canada has focused on large urban settings, specifically in Ontario and British Columbia, making it difficult to generalize the findings nationally and estimate how many 2SLGBTQ+ individuals are experiencing homelessness [2]. The 2018 federal Survey of Safety in Public and Private Spaces of Canadians aged 15 and older found that 2SLGBTQ+ Canadians were more than twice as likely as non-2SLGBTQ+ Canadians to have experienced some type of homelessness or housing insecurity in their lifetime (27% vs. 13%, respectively) [3]. In recent years, a growing body of research has examined 2SLGBTQ+ youth and young adult homelessness across North America; a significantly overrepresented group among youth experiencing homelessness, constituting up to 40% of homeless youth [4–7]. Chapin Hall reported that 2SLGBTQ+ youth are 120% more likely to experience homelessness compared to non-2SLGBTQ+ youth [8]. Identity-based family conflict resulting from a young person coming out as 2SLGBTQ+ is the leading cause of homelessness among 2SLGBTQ+ youth [4, 5, 9]. Previous studies have also reported that family conflict and rejection are predictors of mental health issues among 2SLGBTQ+ individuals [10–12] Similarly, discrimination, exclusion, and stigma contribute to the increased likelihood of poor mental and physical health outcomes among 2SLGBTQ+ youth and young adults experiencing homelessness [4–6].

While research on homelessness has made significant progress in recent years, the experiences of 2SLGBTQ+ individuals facing homelessness have often been overlooked or underrepresented. Accurate prevalence rates are dependent on numerous factors, such as collecting inclusive data and ensuring that sexual orientation and gender identity questions are included on homeless count surveys. Traditional approaches to homelessness tend to generalize the needs and experiences of this diverse population, failing to address the unique challenges they face. Therefore, there is an urgent need for in-depth studies that center on the specific issues affecting 2SLGBTQ+ individuals experiencing homelessness. Toronto, Ontario, Canada, a city known for its diverse population, offers an ideal context to examine the issue of homelessness among 2SLGBTQ+ individuals.

Point-in-time (PiT) counts, also known as “homeless counts”, “street counts”, and “Street Needs Assessments”, are considered the “gold standard” enumeration method to identify the number of people experiencing homelessness at a given time, in a given geographic region or municipality, and to collect demographic and service needs data to better understand the population of people experiencing homelessness. PiT counts also provide an important opportunity to enumerate the prevalence of 2SLGBTQ+ individuals experiencing homelessness and the specific challenges they face accessing stable housing opportunities. If repeated over time, PiT counts can also be used to observe changes in the incidence of homelessness and demographics of people experiencing homelessness in a specific region or municipality. PiT counts generally involve observational data on unsheltered people, administrative data from shelters and transitional housing programs, and surveys of people who are unsheltered or accessing services at a community organization. How and whether demographic data on sexual orientation and gender identity (SOGI) are included in PiT counts are highly inconsistent and often unclear, with many PiT counts categorizing gender/sex as a binary variable (either male/female or male/other) and/or not including sexual orientation data (for representative examples, see: [13–17]. SOGI questions are essential because they represent important aspects of people’s lives and, in many cases, they are directly linked to people’s pathways into and out of homelessness. People’s social identities and locations are frequently related to their pathways into and experiences of homelessness (i.e., through identity-based marginalization) and should be included in any survey which seeks to better understand and ultimately end homelessness.

In Canada, numerous communities have been conducting independent PiT counts for decades. For example, the City of Toronto conducted their first PiT count in 2006 [18]. However, one difficulty in comparing PiT counts is that methodology, including the definition of homelessness used and how demographics such as SOGI are included, can vary significantly between different counts. The Government of Canada’s Reaching Home (Homelessness Strategy) began facilitating coordinated PiT counts across the country in 2016 to improve the utility of PiT count data in Canada [19]. This initiative provided consistent standards, methods, and survey items to be used across all communities. Three such coordinated PiT counts have been conducted thus far, collecting data in 32 communities in 2016, 61 communities in 2018, and 66 communities in 2020–2022 [18, 20, 21].

The inclusion of SOGI questions in Canadian PiT counts have evolved over time. In 2016, the Canadian coordinated PiT count included only one item on gender identity, with <1% providing a response other than male or female and reports only including additional information for binary genders [20]. An additional item on sexual orientation was added in 2018, allowing further analysis. In 2018, 11% of respondents across all PiT counts identified as 2SLGBTQ+ and 2% identified as gender diverse, with youth more likely to identify under the 2SLGBTQ+ umbrella [18]. Preliminary findings from the 2020–2022 national PiT count are similar, with 2% of respondents identifying as gender diverse and 24% of youth identifying as 2SLGBTQ+, a rate that decreases with age [21].

The City of Toronto’s PiT count and survey, the Street Needs Assessment (SNA), has evolved in a similar way. The evolution of these questions has been aimed at developing a more inclusive survey process and better collecting data on 2SLGBTQ+ individuals experiencing homelessness. For a more thorough overview of the evolution of SOGI questions in the SNA surveys see S1 Appendix. In 2006 and 2009, only a single question on gender identity was included, with a new question added in 2013 that asked respondents whether they identify as 2SLGBTQ+. Toronto became a participating community of the national coordinated PiT count in 2018, and as such, adopted the same two SOGI questions presented on the national PiT count survey. The 2021 SNA included a gender identity question (allowing respondents to select one or multiple identities from an expansive list of options), a sexual orientation question (also multiple select), and a question asking whether respondents identified as 2SLGBTQ+.

As Table 1 illustrates, improvements to the SNA survey methodology over the years have generated more accurate data on the size of and specific issues affecting 2SLGBTQ+ individuals experiencing homelessness. Accurate data collection on SOGI is critical to ensure appropriate service provision, as the SNA directly informs the City of Toronto’s Shelter, Support and Housing Administration Homelessness Solutions Service Plan and policy and program development.

**Table 1:** Percentage of all respondents and youth respondents identifying as 2SLGBTQ+ in SNA surveys 2013–2021.

SNA Survey Year	% of 2SLGBTQ+ Respondents	% 2SLGBTQ+ Youth (16–24) Respondents
2013 SNA	9%	21%
2018 SNA	11%	24%
2021 SNA	12%	26%

This manuscript aims to utilize the rich data generated by the most recent City of Toronto SNA survey conducted in 2021 (during the COVID-19 pandemic), to explore the prevalence, causes, experiences, and characteristics of 2SLGBTQ+ individuals experiencing homelessness in Toronto. To date, a relatively small portion of PiT count data has been presented in peer-reviewed publications globally. There has been limited analysis of Canadian PiT count data in peer-reviewed publications, and no analysis of 2SLGBTQ+ individuals in PiT count data. This manuscript draws on a sample of 2SLGBTQ+ respondents (*n* = 288) from the 2021 SNA. It provides recommendations for improving data collection on 2SLGBTQ+ homelessness using large scale surveys, and emphasizes how better data collection can improve homelessness services and housing supports delivery for 2SLGBTQ+ individuals.

## Methods

This article utilizes anonymous secondary data (SNA survey data) collected and analyzed by the City of Toronto, Shelter, Support and Housing Administration (SSHA). The SNA is a needs assessment survey and PiT count of people experiencing homelessness across the city of Toronto led by SSHA, the City division responsible for managing and administering the homelessness service system in Toronto, in collaboration with community partners in the homelessness sector. Surveys were developed through consultation and input from an SNA steering committee comprised of SSHA divisional staff, community partners and advisory boards including the Toronto Alliance to End Homelessness, the Toronto Indigenous Community Advisory Board, and the Toronto Shelter Network and community partners. The survey also underwent review and approval by the City of Toronto Corporate Information Management Services unit, which leads information management policy and implementation and advises on privacy compliance, information security, and best practices and standards for information management. The main objectives of the SNA are to determine the scope and profile of people experiencing homelessness across Toronto; provide people with an opportunity to identify the types of supports and services they need to exit homelessness; and to collect and share critically important data to improve services and programs for people experiencing homelessness in Toronto.

In order to ensure consistency and comparability, the 2021 SNA was held at approximately the same time of year as previous surveys. The indoor survey took place over a one-week period from April 19 to 23, 2021 and was conducted by staff at City-administered emergency and transitional shelters, 24-hour respite sites, 24-hour women’s drop-ins, and COVID-19 response and recovery/isolation sites, and in the Violence Against Women (VAW) shelters which are administered by the Province of Ontario. Any individual staying overnight who was 16 years of age or older was eligible to complete the survey. Due to the COVID-19 pandemic, surveys were not conducted in health and treatment facilities, or correctional facilities. The outdoor survey took place on April 27, 2021 and was conducted by outreach staff from the City of Toronto’s Streets to Homes directly operated and funded outreach agencies. Surveys were administered using an online survey tool and telephone interpretation and survey support was made available at select sites that had resource constraints associated with the COVID-19 pandemic and required additional support to meet survey targets. Unlike in previous surveys, where volunteers were recruited, the 2021 survey was led exclusively by City and community partner agency staff.

Survey site selection was non-random, with all City-administered shelter sites eligible to participate. Almost all (98%) sites participated in the indoor survey. Sites were provided with a survey target for completion during the indoor survey week, which was approximately 50% of their capacity. Outdoor surveys were conducted by outreach staff in areas where people experiencing homelessness are known to be staying. The overall response rate for the 2021 SNA was 45%, with response rates highest in the City’s shelter system and low participation rates in Violence Against Women shelters (see Table 2).

**Table 2:** Response rates for surveyed groups.

Surveyed Groups	2021 Response Rate	Number of Surveys Completed	Total Eligible Population
Outdoors	32%	145	450
City Administered Sites	46%	2437	5305
VAW Shelters	40%	47	117
Total	45%	2629	5872

For the purposes of the SNA survey, homelessness is defined as any individual who, on the night of the survey, was either sleeping outdoors or staying in City-administered emergency/transitional shelters and shelter motels/hotels (including COVID-19 response sites and isolation/recovery programs), 24-hour respite sites (including 24- hour women’s drop-ins), or in Violence Against Women shelters. The SNA does not include people who are experiencing “hidden” homelessness, such as people who are temporarily staying with others (e.g., couch surfing). Community partners have noted that this definition underestimates certain groups such as Indigenous people (notably women and families), 2SLGBTQ+ people, and people with disabilities, all of whom are more likely to experience hidden homelessness. As such, these groups may be underrepresented in the overall results reported.

When researching hard-to-reach populations, including people experiencing homelessness, it is especially important to acknowledge the risk of language bias [22]. Questions used to survey people experiencing homelessness can often be sensitive in nature and triggering for respondents. The questions and response options that are included on surveys and the nature in which questions are asked are critical in ensuring people’s experiences are accurately reflected [23]. For example, including a range of inclusive questions and response options on the PiT count survey helps ensure that respondents see themselves reflected in the questions and that accurate data is collected. Additionally, the survey was translated into French for one of the Francophone VAW shelters and telephone interpretation was used at sites where a need for interpretation services had been pre-identified.

All participants involved in conducting the SNA survey and count were required to complete a series of online training on survey basics and methodology. An Indigenous Cultural Safety Training was developed to help staff better understand the lived experiences of Indigenous people experiencing homelessness. In addition, an online training "Creating an LGBTQ2S Inclusive and Affirmative Street Needs Assessment’’ was delivered by Dr. Alex Abramovich, which focused on definitions of gender identity and sexual orientation, housing challenges affecting 2SLGBTQ+ individuals, and how to ask about gender identity and sexual orientation in respectful, inclusive, and affirming ways. These trainings were aimed at ensuring participant safety in the research process, facilitating researcher’s understanding of the challenges affecting specific marginalized groups, and improving the overall quality of data collection.

### Ethics

The survey underwent review and approval by the City of Toronto Corporate Information Management Services unit, which leads information management policy and implementation and advises on privacy compliance, information security, and best practices and standards for information management. This article utilizes anonymous secondary data from surveys originally collected and analyzed by municipal government housing administration for the purpose of guiding policy and program development, and is thus exempt from requiring research ethics approval.

## COVID-19 context

The COVID-19 pandemic led the City of Toronto to make significant changes to how homeless services are provided and utilized. Collaborating with multiple government bodies, healthcare sectors, and community non-profit organizations, the City responded to the crisis by establishing temporary shelter locations, implementing infection control measures, conducting testing and isolation protocols, prioritizing vaccinations, and focusing on permanent housing solutions. These efforts aimed to mitigate the risks of COVID-19 for homeless individuals who are particularly vulnerable to severe illness. To ensure the safety of all participants involved and comply with public health measures, the approach for the 2021 SNA was modified accordingly. The City’s municipal health department was consulted during the planning and implementation process to ensure adherence to guidelines. The federal government provided communities with adaptation strategies and mitigation measures to conduct the PiT counts safely based on the level of risk and severity of the pandemic’s impact. Due to restrictions on non-essential visitors in shelter locations, volunteers were not recruited, and surveys were primarily led by staff from City-administered shelters, outreach agencies, and provincially-administered sites. All survey training was conducted online, and surveys were administered electronically to minimize in-person contact.

## Data collection

The SNA surveys were administered to clients by trained shelter and outreach staff using a computer or mobile device. Verbal informed consent was obtained from each participant prior to beginning the survey; participants were informed that participation was voluntary and would not affect their services. Verbal consent, or lack thereof, was recorded in the Shelter Management Information System to ensure participants were not approached or surveyed more than once. No names or identifiers were attached to individuals’ survey responses. A $10 honoraria was provided to participants upon completion of the survey.

The survey took approximately 10–15 minutes to complete and included questions on demographic characteristics, living situation, experiences of homelessness, reasons for homelessness, service use, health challenges, and substance use treatment. To assess gender identity, participants were asked and shown the question: “*What gender do you identify with*”, with the following response options: *Male/Man*, *Female/Woman*, *Trans Male/Trans Man*, *Trans Female/Trans Woman*, *Two-Spirit*, *Non-Binary (Genderqueer)*, *Don’t know*, *Decline to answer*, and *Not Listed* (with free text response). To assess sexual orientation, participants were asked and shown the question: “*How do you describe your sexual orientation*, *for example straight*, *gay*, *lesbian*?”, with the following response options: *Straight/Heterosexual*, *Gay*, *Lesbian*, *Bisexual*, *Two-Spirit*, *Pansexual*, *Asexual*, *Questioning*, *Queer*, *Don’t know*, *Decline to answer*, and *Not listed* (with free text response). To ensure that 2SLGBTQ+ respondents saw themselves reflected in the survey, an additional question was included stating: “*Do you identify as LGBTQ2S+ (lesbian*, *gay*, *bisexual*, *transgender*, *queer*, *questioning*, *2-spirit*, *+ refers to sexual and gender diverse identities not represented in the acronym)*?” with the following response options: *Yes*, *No*, *Don’t Know*, *Decline To Answer*.

## Analysis

Anonymous data from the 2021 Street Needs Assessment was accessed on February 23, 2023. All data analysis was conducted by City of Toronto, Shelter, Support and Housing Administration staff (CW, LW). All other authors viewed aggregate data only and did not have access to participant-level survey responses.

Data analysis was conducted in Microsoft Excel using descriptive statistics, specifically frequencies, distributions, and proportions. Respondents were stratified by 2SLGBTQ+ individuals and non-2SLGBTQ+ individuals, and further stratified by age categories including youth (16–24), adults (25–58), and older adults (60+).

There are a number of ways to calculate the size of the 2SLGBTQ+ population in the survey data. One method is to use affirmative responses to the question “*Do you identify as LGBTQ2S+*?*”* By this measure 10% of all survey respondents (*n* = 233) identified as 2SLGBTQ+. Another method is to create a derived variable using Sexual Orientation responses other than *Straight/Heterosexual* and gender identities other than *Man* or *Woman*. By this measure 12% (n = 288) of all survey respondents identified as 2SLGBTQ+. The derived variable method produces a more accurate sample of this population and includes those who may not feel comfortable stating directly that they identify 2SLGBTQ+. It is worth noting, however, that participants who identified as Trans man and/or Trans woman may also identify as man and/or woman (using the multiple select gender identity options) resulting in some degree of over counting among these individuals. In the analysis that follows, we used the derived variable of *n* = 288 as it provides a more comprehensive and inclusive sample for data interpretation.

## Results

### Participants

The sociodemographic characteristics of the study sample are found in Table 3. The average age of 2SLGBTQ+ respondents was 34 years, which is younger than non-2SLGBTQ+ respondents’ average age of 46 years. More than one-third of participants identified their sexual orientation as bisexual and their gender identity as woman. Participants also described their sexual orientation as gay, lesbian, pansexual, asexual, questioning, and queer. Additional gender identities reported by participants include man, transgender woman, transgender man, Two-spirit, and non-binary. Because participants were able to select multiple gender identity and sexual orientation categories, some participants identified with two, three, or four gender identities and sexual orientations. A quarter of participants (25%; *n =* 72) reported that they had been in foster care or a group youth home in the past, compared to 17% of non-2SLGBTQ+ respondents.

**Table 3:** Sociodemographic characteristics of sample (n = 288).

Age Category	*n (%)*
16–24	81 (28%)
25–56	189 (66%)
60+	18 (6%)
**Gender Identity** (multiple responses permitted)	
Female/Woman*	124 (41%)
Male/Man*	96 (31%)
Transgender Female/Transgender Woman	25 (8%)
Transgender Male/Transgender Man	8 (3%)
Gender Diverse (Two-spirit, non-binary, genderqueer)	44 (15%)
Do not know	6 (2%)
Decline to answer	<5(1%)
**Sexual Orientation** (multiple responses permitted)	
Asexual	22 (7%)
Bisexual	116 (34%)
Gay	58 (17%)
Lesbian	32 (9%)
Pansexual	28 (8%)
Queer	13 (4%)
Questioning	16 (5%)
Straight/Heterosexual	33 (10%)
Two-spirit	17 (5%)
Decline to answer	<5 (1%)
**Racial Identity** (multiple responses permitted)	
Arab	11 (3%)
Asian (East, South, Southeast, West) or Indo-Caribbean	39 (12%)
Black/African/Caribbean	98 (28%)
First Nations (with or without status), Métis, or Inuit	55 (16%)
Latin American	17 (5%)
White/European	122 (36%)
**Immigration Status**	
Born in Canada	201 (70%)
Immigrant	39 (14%)
Refugee Claimant	25 (9%)
Refugee	12 (4%)
Temporary Resident	6 (2%)
Do not know	<5 (1%)
Decline to answer	<5 (0)%

*Note. Cisgender was not specified.

Participants reported diverse racial identity backgrounds. Notably, 64% of participants identified as racialized with 16% identifying as First Nations, Métis, or Inuit. Participants were also asked about immigration and refugee status. The majority of 2SLGBTQ+ individuals (70%) identified as being born in Canada; 14% of participants were immigrants, and 13% were refugees or refugee claimants. These proportions differed significantly from the 2018 SNA survey, where 48% of 2SLGBTQ+ respondents were born in Canada, 12% were immigrants, and 28% were refugees or refugee claimants. These variations are likely due to the lower overall number of refugees in the shelter system in 2021 due to border restrictions implemented by the federal government in response to the COVID-19 pandemic.

### Education, income, and family status

When asked about their level of education, 24% (*n* = 69) of respondents reported completing high school, 19% (*n* = 54) completed a post-secondary degree, and 3% (*n* = 8) completed a graduate degree (e.g., Master’s, PhD). Additionally, 18% (*n* = 52) reported having some post-secondary education, 31% (*n* = 87) reported completing some high school, 2% (*n* = 7) reported completing primary school, and less than 1% (*n* = <5) reported having no formal education.

Data was also collected on participant’s primary sources of income. The majority (61%) of participants reported their main source of income from social assistance (Ontario Works or Ontario Disability Support Program). Other government support programs reported include GST Refund (4%; *n* = 14), Employment Insurance (2%; *n* = 7), Seniors Benefit (2%; *n* = 7), Canada Child Benefit (2%; *n* = 7), Veteran/VAC benefits (>1%; *n* = >5), and other service agencies (3%; *n* = 9). Participants also reported various forms of employment as their main source of income, including casual employment (3%; *n* = 9), part-time employment (4%; *n* = 16), and full-time employment (3%; *n* = 11). Additionally, 4% (*n* = 13) reported receiving money from friends and/or family, while 2% (n = 8) reported receiving income from other informal income services. Notably, 8% (*n* = 27) reported having no income. See Table 4 for a description of income sources.

**Table 4:** Sources of income reported by 2SLGBTQ+ respondents.

Income Source	*n* (%)
Ontario Works	114 (32%)
Ontario Disability Support Program	103 (29%)
No Income	27 (8%)
GST Refund	14 (4%)
Money from Friends/Family	13 (4%)
Part Time Employment	16 (4%)
Full Time Employment	11 (3%)
Casual Employment	9 (3%)
Other	11 (3%)
Other money from Service Agency	9 (3%)
Canada Child Benefit	7 (2%)
Employment Insurance	7 (2%)
Informal Income Services	8 (2%)
Seniors Benefit (CPP/OAS/GIS)	7 (2%)

Respondents were asked which family members (if any) they were staying with at the time they completed the survey (e.g., children/dependents, partner) as a measure of family status. The vast majority of participants (87%; *n* = 250) reported staying alone, 8% (*n* = 23) reported staying with a partner, 4% (*n* = 13) reported staying with a family head and dependents, and 1% (*n* = <5) reported staying with another adult.

### Length of time homeless

Both the Government of Canada and the Province of Ontario have prioritized ending chronic homelessness, which is defined for the purposes of PiT counts as an experience of homelessness lasting for six months or more in the past year. 2SLGBTQ+ respondents experienced high rates of chronic homelessness, with 77% (*n* = 224) reporting chronic homelessness in the past year (Table 5).

**Table 5:** Amount of time homeless in the last 12 months and over lifetime.

Time Homeless in the Last 12 Months	*n (%)*
12 Months	176 (60%)
10–11 Months	7 (2%)
7–9 Months	24 (8%)
4–6 Months	41 (14%)
1–3 Months	31 (11%)
Do not know	8 (3%)
Decline to answer	<5 (1%)
**Homelessness Over Lifetime**	***n (%)***
Entire Life	10 (3%)
More than 10 years	18 (6%)
6–10 Years	39 (14%)
1–5 Years	133 (47%)
<1 Year	59 (21%)

Almost half (47%; *n* = 133) of participants had experienced homelessness for a period of 1–5 years over their lifetime, 21% (*n* = 59) had experienced homelessness for less than a year, 14% (*n* = 39) reported between 6–10 years, 6% (*n* = 18) had experienced homelessness for more than 10 years, and 3% (*n* = 10) had experienced homelessness for their entire life. See Table 5 for length of time homeless over lifetime and within the last 12 months.

### Overnight locations

**Fig 1:**
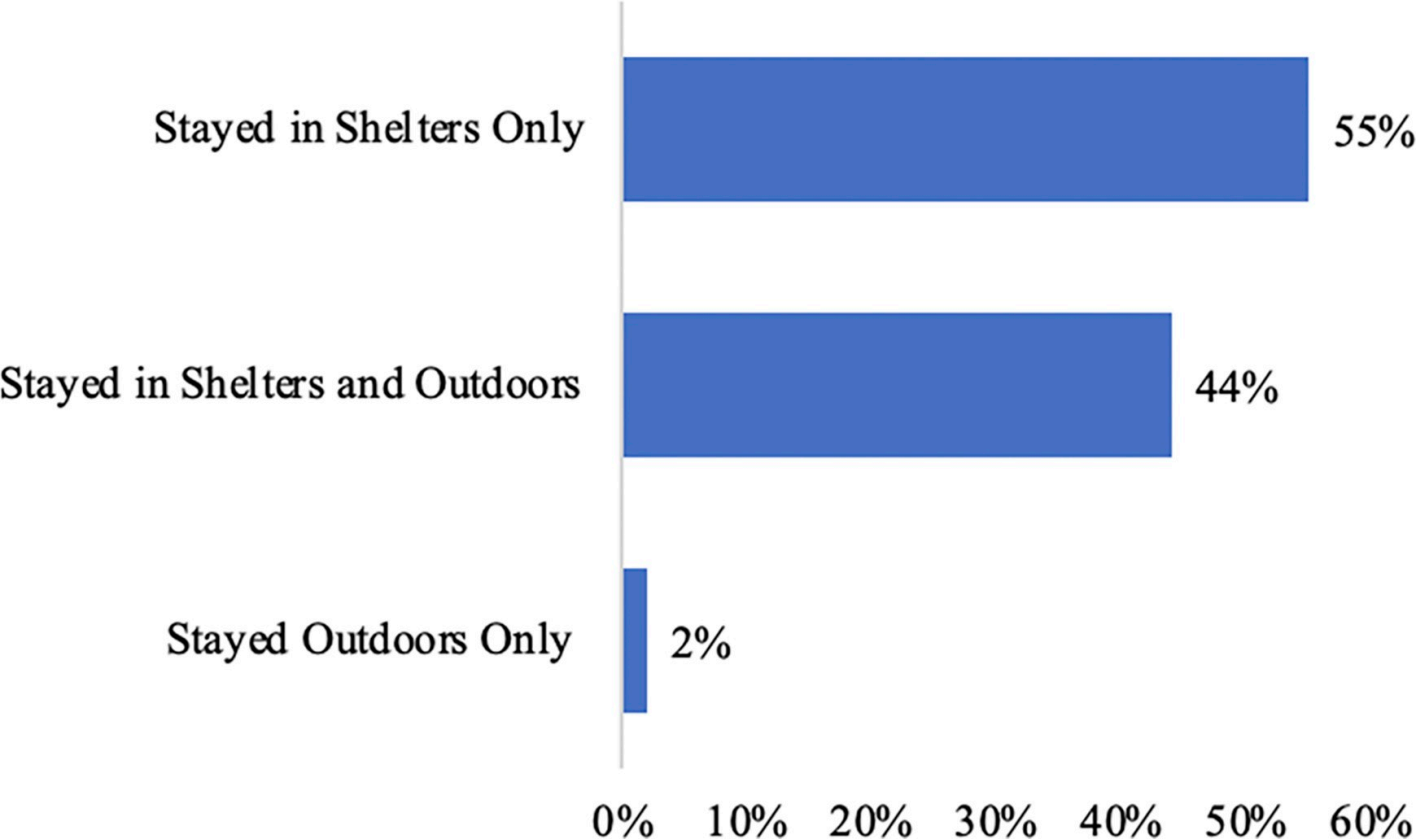
Movement between indoor and outdoor locations.

At the time of the survey, the vast majority of 2SLGBTQ+ respondents (95%; *n* = 274) were staying in City-administered shelters, with 5% (*n* = 14) staying in outdoor locations. Participants were also asked to list where they had stayed in the past year. Over half of respondents (55%; *n* = 138) reported that they stayed exclusively in shelters over the past year, while 44% (*n* = 110) stayed in shelters and outdoors, and 2% (*n* = >5) reported that they stayed exclusively outdoors (see Fig 1). Among those who had stayed outdoors in the past year, 44% (*n* = 126) reported staying in an outdoor location (e.g., a laneway, abandoned building, ravine, vehicle, park, or bus shelter), and 21% (*n* = 60) reported staying in an encampment (defined as an outdoor area where an individual or a group of people live in homelessness together, often in tents or other temporary built structures).

### Pathways into homelessness

The average age that 2SLGBTQ+ respondents first experienced homelessness was 26 years, which was significantly younger than non-2SLGBTQ+ respondents at 33 years. The majority of 2SLGBTQ+ respondents first reported experiencing homelessness as youth, with 60% experiencing homelessness before they were 24 years old. By comparison, 32% of non-2SLGBTQ+ respondents experienced homelessness before they were 24 years old.

**Fig 2:**
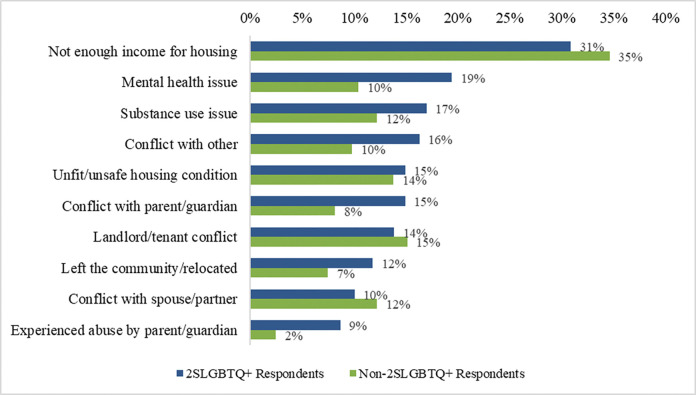
Reasons for housing loss reported by 2SLGBTQ+ and non-2SLGBTQ+ respondents.

Respondents reported numerous reasons for their most recent housing loss (see Fig 2). The most frequently reported reason among 2SLGBTQ+ respondents was not enough income for housing (31%; *n* = 89), which was also the most frequent response shared by non-2SLGBTQ+ respondents at 35%. This was followed by mental health issues (19%; *n* = 56), substance use issues (17%; *n* = 49), and conflict with others (16%; *n* = 47). The most notable differences in reasons for housing loss between 2SLGBTQ+ and non-2SLGBTQ+ respondents were mental health issues, conflict, and abuse. 15% of 2SLGBTQ+ respondents reported conflict with a parent/guardian compared to 8% of non-2SLGBTQ+ respondents. Relatedly, 9% of 2SLGBTQ+ respondents reported that they experienced abuse by a parent/guardian, compared to 2% of non-2SLGBTQ+individuals. Mental health issues were common pathways into homelessness among 2SLGBTQ+ respondents, and were also identified as a significant health issue, with 73% (*n* = 210) reporting a mental health issue, notably higher than 50% of non-2SLGBTQ+ respondents. Among 2SLGBTQ+ youth, conflict with parent/guardian was the leading reason for housing loss at 41% (*n* = 33) followed by experienced abuse by parent/guardian at 25% (*n* = 20). A significant number of youth (63%; n = 51) reported conflict with a parent/guardian, partner or other as a major reason for housing loss. Additional reasons for housing loss reported by 2SLGBTQ+ youth included not enough income for housing (26%; *n* = 21); mental health issues (15%; *n* = 12); and discrimination (15%; *n* = 12).

The SNA survey also included questions on whether respondents had always lived in Toronto and if not, where they had lived prior to moving to the city. Over half of respondents (61%; *n* = 177) reported living elsewhere prior to coming to Toronto, whereas 37% (*n* = 107) reported always having lived in Toronto. Among the respondents who had lived elsewhere, 31% (*n* = 91) reported that they were previously living in another community in Ontario, with the majority coming from Mississauga, Brampton, and Ottawa; 15% (*n* = 42) reported that they came to Toronto from another province in Canada, with British Columbia, Quebec, and Alberta as the leading provinces; and 15% (*n* = 44) of respondents came to Toronto from another country, with the majority coming from Mexico, Jamaica, and the United States.

Among those who had lived elsewhere, 64% (*n* = 117) became homeless after arriving in Toronto, while 34% (*n* = 62) were homeless prior to arriving in Toronto. Within this group, 73% (*n* = 47) had used shelter services in their previous community while 23% (*n* = 15) had first accessed shelter services when they came to Toronto. 2SLGBTQ+ individuals who moved to Toronto provided distinct reasons for moving to the city. As Table 6 demonstrates, the leading reasons were to access services and supports, to seek employment, fear for safety, to find housing, and to attend school. 2SLGBTQ+ respondents were nearly twice as likely to report ‘fear for safety’ as a reason for moving to Toronto compared to non-2SLGBTQ+ respondents.

**Table 6:** Top ten reasons 2SLGBTQ+ respondents moved to Toronto.

Top Ten Reasons for Moving to Toronto	*n* (%)
To access services and supports	45 (15%)
To seek employment	42 (14%)
Fear for safety	38 (13%)
To find housing	33 (11%)
To attend school	27 (9%)
Family moved here	26 (9%)
To visit friends/family	26 (9%)
To access emergency shelter(s)	23 (8%)
Other reasons	22 (7%)
To be closer to cultural supports and connections	17 (6%)

For participants who lived elsewhere prior to coming to Toronto, the reasons for housing loss were slightly different than respondents who had always been in Toronto. For the former, the top two reasons for housing loss were also not enough income (27%; *n* = 49) and conflict with and/or abuse by parent/guardian (19%; *n* = 35), similar to those who had always lived in Toronto. However, the third most common reason for housing loss among those who lived elsewhere prior to Toronto included general conflict with others (17%; *n* = 31). Further, leaving community/relocation was also among the top four reasons for housing loss (16%; *n* = 30) for those who had moved to Toronto. See Table 7 for reasons for housing loss amongst those who lived elsewhere before moving to Toronto.

**Table 7:** Primary reasons for housing loss for those who lived elsewhere prior to moving to Toronto.

Reason for Housing Loss	*n* (%)
Not enough income for housing	49 (27%)
Conflict with and/or abuse by parent/guardian	35 (19%)
Conflict with other	31 (17%)
Left the community/relocated	30 (16%)
Conflict with and/or abuse by spouse/partner	30 (16%)
Mental health issue	27 (15%)
Unfit/unsafe housing condition	25 (14%)
Landlord/tenant conflict	25 (14%)
Substance use issue	24 (13%)
Other	19 (10%)

## Discussion

This is the first study in Canada to utilize PiT count data to analyze 2SLGBTQ+ individuals experiencing homelessness. By including specific SOGI questions in this survey, researchers are better able to understand the experiences, needs, and challenges among 2SLGBTQ+ individuals experiencing homelessness in Toronto. Understanding these experiences is critical, as 2SLGBTQ+ individuals are disproportionately represented among those experiencing homelessness, and research findings can help inform changes to both policy and programming that will directly impact 2SLGBTQ+ individuals.

Consistent with previous research, our study found that in comparison with non-2SLGBTQ+ individuals experiencing homelessness, 2SLGBTQ+ respondents were younger at the time of the survey, were younger when they first experienced homelessness, were more likely to have been in foster care or a group home, and were more likely to report conflict with and/or abuse by a parent/guardian as their main pathway into homelessness. Identity-based family rejection resulting from a young person coming out as 2SLGBTQ+ is a major contributing factor to youth homelessness and the most frequently cited pathway into homelessness among 2SLGBTQ+ youth in North America [5], a finding that is echoed in the Toronto SNA results. Previous research has reported that risks associated with family violence and abuse are elevated among 2SLGBTQ+ youth, including coming out as 2SLGBTQ+ resulting in familial conflict, abuse, and being kicked out of the house or being forced to leave home, due to safety concerns [4, 5, 9, 24]. Being unhoused at a younger age can have a major impact on one’s life trajectory. For example, being unstably housed while still in school can negatively impact a person’s focus and performance, resulting in poor mental health outcomes and difficulties completing their education [5, 25].

2SLGBTQ+ individuals also reported high rates of chronic homelessness, with over three-quarters of those surveyed reporting periods of homelessness lasting 6 months or more in the past year. Research has found that individuals who experience homelessness at younger ages tend to experience multiple episodes of homelessness, be involved with child welfare and protection services, experience bullying, have higher rates of mental health and substance use issues, experience suicidality, and become chronically homeless, resulting in challenges and barriers successfully exiting homelessness [26].

When youths’ source of food and shelter are precarious, securing formal employment to maintain these essentials is a priority, even if this impedes their ability to continue their education. However, without formal education, the limited employment opportunities available often pay less than a living wage, restricting the affordability of housing options. Our data shows that 33% of participants moved to Toronto to seek employment and housing, including emergency shelter (see Table 6), 27% of participants reported their reason for housing loss was “not enough income” (Table 7), and 61% of participants reported government assistance as their main source of income (Table 4).

Consistent with previous studies, which have found that racialized 2SLGBTQ+ individuals are more likely to experience poverty and discrimination, our findings report that a high proportion of 2SLGBTQ+ individuals experiencing homelessness in Toronto are racialized. The marginalization that occurs at the intersection of race, sexual orientation, and socioeconomic status is evident in the SNA results. For example, 22% of 2SLGBTQ+ participants completed post-secondary education and reported not having enough income for housing (Table 7), highlighting how education does not sufficiently buffer the impact of experiencing homelessness and navigating services with multiple marginalized identities. Further, 2SLGBTQ+ individuals reported high rates of mental health issues, reflecting the unique mental health challenges faced by 2SLGBTQ+ individuals experiencing homelessness who are subjected to stigma, racism, discrimination, and victimization [27]. Research suggests that 2SLGBTQ+ individuals experience additional barriers when seeking support from healthcare providers [28] and that gender identity and sexual orientation are social determinants of mental health [29]. Lastly, 2SLGBTQ+ individuals frequently report feeling unsafe in housing programs due to stigma and discrimination, resulting in higher rates of hidden homelessness [5].

The vast majority of 2SLGBTQ+ respondents reported that they were residing in City-administered shelters at the time of the survey. This aligns with the findings in Table 6, which highlight that 2SLGBTQ+ respondents reported moving to Toronto to access services, given that 2SLGBTQ+ population-based services are typically located in metropolitan areas. Housing programs, including emergency shelters, should be located in peoples’ communities (urban, rural, suburban) to avoid the need to relocate and leave behind important social connections, which has been linked to worsened health, fewer social networks, and increased risk of victimization [30, 31]. It is imperative that existing programs serving people experiencing homelessness commit to and take the necessary steps to ensure that programs are 2SLGBTQ+ inclusive and safe by providing mandatory 2SLGBTQ+ inclusion training to all staff, respecting and accepting people’s pronouns and chosen name, ensuring the availability of all-gender washrooms, and having an up-to-date list of 2SLGBTQ+ affirming resources.

The overall response rate for the 2021 SNA was 45%, with response rates highest in the City’s shelter system and low participation rates in Violence Against Women shelters. The average response rates for previous City of Toronto, SNAs include: 2013, 40% response rate, and 2018, 51% response rate. The lower response rate was mainly due to outbreaks occurring as the 2021 SNA was conducted during the peak of the third wave of the COVID-19 pandemic. Additionally, volunteers were not recruited for the 2021 SNA, and surveys were primarily led by staff from City-administered shelters, outreach agencies, and provincially-administered sites. Despite this, the sample size utilized for the surveyed population results in a 99% confidence level with a 2% margin of error.

There are numerous reasons that 2SLGBTQ+ individuals may not have participated in the SNA survey, including safety concerns, stigma, and discrimination, which are also factors that keep 2SLGBTQ+ people from accessing services. The PiT count methodology only captures people experiencing homelessness who are staying in overnight shelter spaces, staying outdoors or in health and treatment facilities or correctional facilities and does not include people who may be experiencing hidden homelessness—which many 2SLGBTQ+ youth are.

The authors speculate that outdoor response rates (32%) may be lowest due to lower approach rates and perceived safety concerns on behalf of survey administrators in conjunction with the willingness of people to fill out a survey after being approached, in the areas they are sleeping. Outreach staff noted that people staying outdoors were less interested in participating in the survey due to the rain. Additionally, while the outdoor response rates are the lowest, they are consistent with previous years (e.g., in 2018, the outdoor response rate was 31%). Based on the sector specific response rates, the average is brought down by the lower response rates in the provincially administered VAW shelters. Only 9/14 (64%) of VAW shelters participated in the survey at their sites. The low participation rate from VAW shelters is mainly due to the majority of them being in COVID-19 outbreak during the SNA. In previous years, the response rates from the VAW shelters were 75%, whereas is in 2021 the response rate was 40%. Further, if peoples’ pathway into homelessness is a result of familial or partner violence there may be an additional level of reluctance to identify as homeless which may impact response rates as well. Lastly, the higher response rates from people accessing city administered sites (46%) may be due to people being more familiar with the staff who are present the night of the survey. Participation may be higher in City-administered sites because the City provides funding to these sites unlike the VAW shelters which are funded and administered by the province. Participation in research and evaluation initiatives may be higher among City-administered sites as they consider it to be a part of their operating agreements.

All participants, regardless of which services they are or are not accessing, may choose not to participate. We speculate that people may not want to be bothered during their evening or may feel their participation reduces them to a statistic. Regardless, people’s right to not participate is respected, and survey administrators are not prompted to pry any further. Importantly, while SNAs and PIT counts aim to enumerate the number of people experiencing homelessness on a given evening, their motivations for participating, or their rationale for refraining, are outside the scope of the study.

The results of this study highlight the necessity of including SOGI questions in PiT count surveys, as there is a significant need to better understand and address the unique experiences, challenges, and needs among 2SLGBTQ+ individuals experiencing homelessness. Improved data collection will help determine how many 2SLGBTQ+ individuals are experiencing homelessness across Canada and confirm important demographic characteristics of the population, resulting in new and improved data on an under researched and largely hidden population. Accurate prevalence rates and data on the needs and challenges among 2SLGBTQ+ individuals experiencing homelessness in communities across Canada will provide guidance on how to improve existing policies and services addressing homelessness and on the development of new responses and strategies for preventing, reducing, and ultimately ending 2SLGBTQ+ homelessness.

### Strengths and limitations of PiT count methods

The PiT count methodology offers many strengths for researching homelessness among 2SLGBTQ+ individuals. Firstly, it is an efficient method for researchers to collect data from a large population within a short timeframe. As such, PiT counts are advantageous when sampling vulnerable populations, such as 2SLGBTQ+ individuals, as it provides real-time information allowing researchers and policymakers to understand the immediate prevalence and needs of these populations. The standardized approach of PiT counts also facilitates and allows comparisons to be made across regions or time periods which is conducive to identifying trends, and patterns that can inform policy and program development.

The methodology developed for the SNA 2021, particularly the inclusion of three SOGI questions also provides lessons for how best to assess the size of 2SLGBTQ+ populations in survey research. The derived variable method employed here is but one technique to capture a more representative and comprehensive sample, and to include those individuals who may not feel comfortable stating that they directly identify as 2SLGBTQ+. Further, the training of research teams prior to data collection suggests that one technique for improving data collection on 2SLGBTQ+ populations is to develop inclusive and affirming survey questionnaires and scripts. For example, reading aloud the gender identity and sexual orientation options listed on the survey may generate feelings of recognition and validation. This process of making individuals ‘feel seen’ increases the likelihood that they will disclose features of their social identity. Surveys such as the SNA are conducted during periods of significant personal stress and dislocation in participants’ lives, ensuring that researchers understand this and can respond appropriately does more than improve the quality of the data, it recognizes the humanity of participants.

Despite their strengths, PiT counts have limitations that must be considered when examining homelessness among 2SLGBTQ+ individuals. The snapshot nature of PiT counts may not capture the dynamic and nuanced experiences of 2SLGBTQ+ individuals over time. Furthermore, the limited representativeness of PiT counts can lead to selection bias as a result underrepresenting certain subgroups within the 2SLGBTQ+ homeless population. There may also be further bias due to the self-reported nature of PiT counts. Additionally, measurement error should be considered. For example, it is possible that some respondents did not feel safe or comfortable coming out as 2SLGBTQ+ on the survey, and some people’s identities are fluid and/or they may identify with more than one label but only selected the one that aligned with their identity on the day of the survey. Some transgender individuals or people with transgender lived experience may identify as a woman or man, rather than as a transgender woman or transgender man, and it is possible that some respondents did not understand the SOGI questions. Additionally, there are reasons that 2SLGBTQ+ individuals may not have participated in the SNA survey, including safety concerns, stigma, and discrimination, which are also factors that keep 2SLGBTQ+ people from accessing services and contribute to the high rates of hidden homelessness. People experiencing homelessness are characterized as a hard-to-reach population, resulting in recruitment challenges for research purposes. Lastly, PiT counts cannot measure hidden homelessness, which is important to note, as 2SLGBTQ+ individuals are more likely to experience hidden homelessness and may be underrepresented in PiT counts. Researchers should be cautious about generalizing results beyond the specific context and be aware of the potential limitations associated with PiT counts in studying 2SLGBTQ+ homelessness.

## Conclusion

The findings from this study demonstrate that Street Needs Assessments and PiT counts can be used to examine homelessness in marginalized populations, including 2SLGBTQ+ individuals and that SOGI questions need to be included on future government surveys. Additionally, the consistency of findings from this study and previous research suggests that 2SLGBTQ+ individuals’ experiences of homelessness are unique and challenging, resulting in a significant need for population-based housing and social support services specifically for 2SLGBTQ+ populations.

## Supporting information

S1 Appendix Overview of SOGI questions in SNA surveys 2006–2021. (TIF)
