# Supplementary figures and images for: Identifying 2SLGBTQ+ individuals experiencing homelessness using Point-in-Time counts: Evidence from the 2021 Toronto Street Needs Assessment survey
Source: PLoS One. 2024 Apr 10;19(4):e0298252. doi: 10.1371/journal.pone.0298252 (PMC11006143; doi:10.1371/journal.pone.0298252)

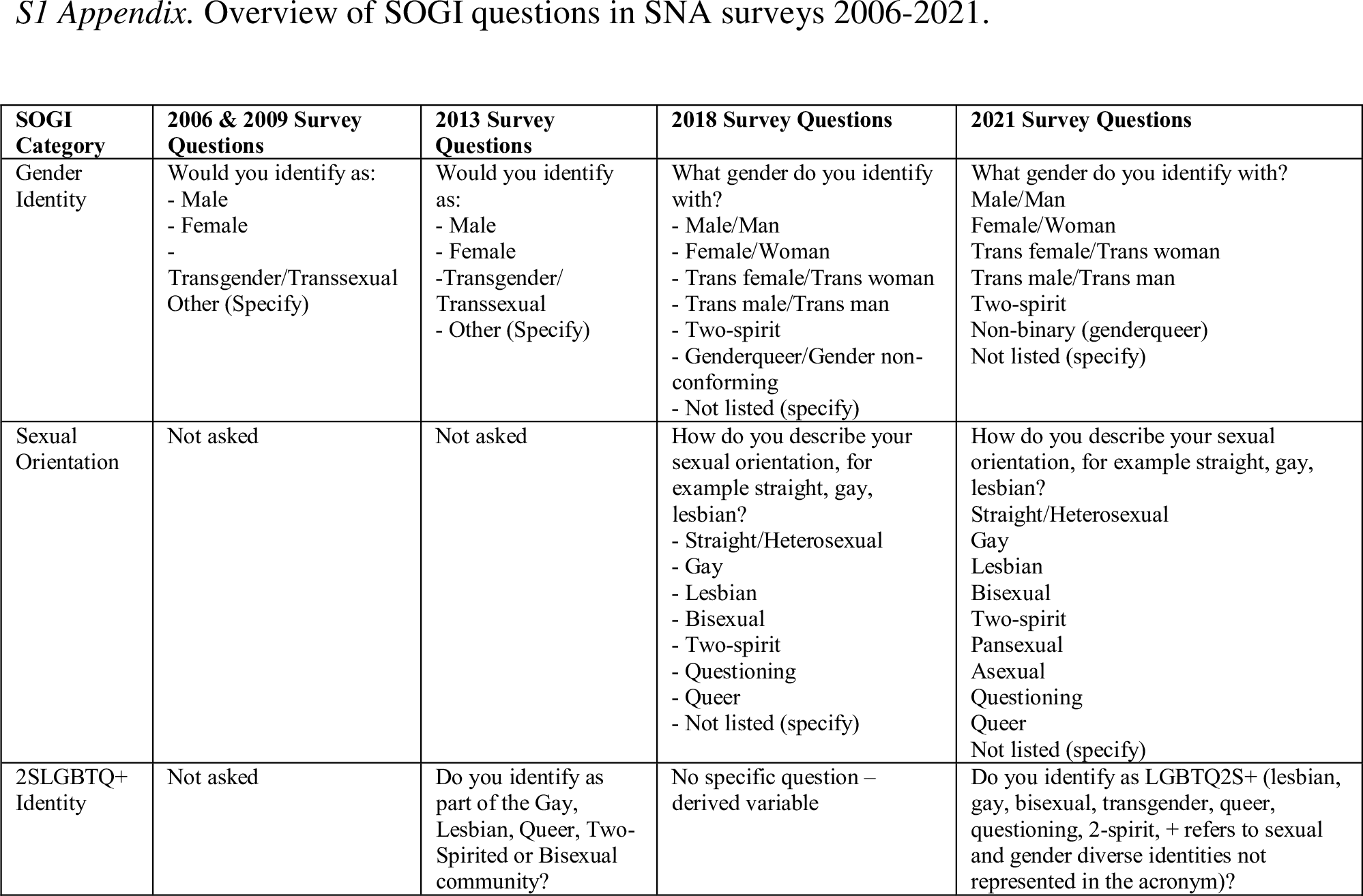

Supplement: S1 Appendix — (TIF) [file pone.0298252.s001.tif]
